# Genome Sequence of *Mannheimia haemolytica* MHA.Sh.MOR19 Serotype 1, a Moroccan Sheep Isolate

**DOI:** 10.1128/MRA.00359-21

**Published:** 2021-05-27

**Authors:** Dounia Bkiri, Siham Fellahi, Slimane Khayi, Khalid Omari Tadlaoui, Ouafaa Fassi Fihri, Mehdi El Harrak

**Affiliations:** a Research and Development Department, Multi-Chemical Industry, Mohammedia, Morocco; b Institute of Agronomy and Veterinary Medicine Hassan II, Rabat, Morocco; c National Institute of Agronomic Research, Rabat, Morocco; Loyola University Chicago

## Abstract

Mannheimia haemolytica is the principle bacterial pathogen in ruminants associated with respiratory disease. Here, we report the draft genome sequence of the Mannheimia haemolytica MHA.Sh.MOR19 strain that was recently isolated in the northwest of Morocco from the lung of a lamb that died from pneumonia. The genome size is 2,434,458 bp.

## ANNOUNCEMENT

Mannheimia haemolytica is one of the most common agents associated with pneumonia in ruminants (1) throughout the world and causes an economically important disease due to associated morbidity, mortality, and treatment costs (2). Mannheimia haemolytica is a Gram-negative bacterium of the upper respiratory tract and nasopharynx of ruminants (3).

Infection due to Mannheimia haemolytica has been rarely described in small ruminants. We recently isolated this bacterium from a dead lamb with characteristic respiratory macroscopic changes. The tissue was enriched immediately after sample collection in tryptone soya broth medium at 37°C for 24 h. The suspension was then inoculated onto solid medium with agar-based medium supplemented with 5% sheep blood and incubated at 37°C for 24 hours under aerobic conditions. Typical colonies with the presence of hemolysis were transferred to be grown in brain heart infusion (BHI) broth for 9 h at 37°C with moderate agitation (4).

Diagnosis of Mannheimia haemolytica infection relies primarily on biological and molecular characterization. PCR targeting specific genes (*Pasteurella haemolytica* serotype 1-specific antigens [PHSSA]) (Rpt2) (5) was used to detect Mannheimia haemolytica in tissues and bacterial culture. Serotyping of this isolate was performed using a PCR assay by amplification of hypothetical protein gene forward (5′-CAT TTC CTT AGG TTC AGC-3′) and reverse (5′-CAA GTC ATC GTA ATG CCT-3′) primers for serotype 1-specific detection (6).

To understand the mechanism of this infection in small ruminants, we proceeded with molecular characterization of Mannheimia haemolytica strain MHA.Sh.MOR19. Genomic DNA was extracted from fresh bacterial culture, using the isolate II genomic DNA kit (Bioline), and resuspended in 100 μl of nuclease-free distilled water. After DNA fragmentation, 500-bp genomic DNA fragments were selected using E-Gel SizeSelect and then sequenced using 2 × 150 bp by Eurofins Genomics using the Illumina MiSeq platform. The raw data were trimmed based on the cutoff low-quality (limit, 0.05) and ambiguous nucleotides (*n* ≤ 2) with CLC genomics v12 (Qiagen, Hilden, Germany). In total, 13,889,863 reads were recovered with an average length of 150 bp. *De novo* assembly was carried out using CLC genomics v12 with default parameters (length fraction, 0.5; similarity, 0.8) that generated 46 contigs (>2,000 bp) with an average coverage of 400×. The largest contig was 216,368 bp and the smallest was 2,057 bp. The *N*_50_ parameter for the contigs was estimated to be 104,821 bp long. The size of the assembled Mannheimia haemolytica MHA.Sh.MOR19 strain is 2,434,458 bp with a G+C content of 41%.

### Data availability.

This whole-genome shotgun project has been deposited at DDBJ/ENA/GenBank under the accession number JAFIRP000000000. The version described in this paper is the first version, JAFIRP000000000.1. The Illumina reads are available in the SRA under accession number SRR14069062.

## References

[1] Taunde PA, Argenta FF, Bianchi RM, de Cecco BS, Vielmo A, Lopes BC, Siqueira FM, de Andrade CP, Snel GGM, Barros CSLD, Sonne L, Pavarini SP, Driemeier D. 2019. Mannheimia haemolytica pleuropneumonia in goats associated with shipping stress. Cienc Rural 49:1–6. doi:10.1590/0103-8478cr20180621.

[2] Ozbey G, Kilic A, Ertas HB, Muz A. 2012. Random amplified polymorphic DNA (RAPD) analysis of Pasteurella multocida and Manheimia haemolytica strains isolated from cattle, sheep and goats. Vet Med 49:65–69. doi:10.17221/5678-VETMED.

[3] Narayanan SK, Nagaraja TG, Chengappa MM, Stewart GC. 2002. Leukotoxins of Gram-negative bacteria. Vet Microbiol 84:337–356. doi:10.1016/S0378-1135(01)00467-9. 11750142

[4] Klima CL, Alexander TW, Read RR, Gow SP, Booker CW, Hannon S, Sheedy C, McAllister TA, Selinger LB. 2011. Genetic characterization and antimicrobial susceptibility of Mannheimia haemolytica isolated from the nasopharynx of feedlot cattle. Vet Microbiol 149:390–398. doi:10.1016/j.vetmic.2010.11.018. 21146332

[5] Legesse A, Abayneh T, Mamo G, Gelaye E, Tesfaw L, Yami M, Belay A. 2018. Molecular characterization of Mannheimia haemolytica isolates associated with pneumonic cases of sheep in selected areas of Central Ethiopia. BMC Microbiol 18:205. doi:10.1186/s12866-018-1338-x. 30518323 PMC6280500

[6] Klima CL, Zaheer R, Briggs RE, McAllister TA. 2017. A multiplex PCR assay for molecular capsular serotyping of Mannheimia haemolytica serotypes 1, 2, and 6. J Microbiol Methods 139:155–160. doi:10.1016/j.mimet.2017.05.010. 28551457

